# Computational Analysis of Mapping Catheter Geometry and Contact Quality Effects on Rotor Detection in Atrial Fibrillation

**DOI:** 10.3389/fphys.2021.732161

**Published:** 2021-12-09

**Authors:** Chiara Bartolucci, Claudio Fabbri, Corrado Tomasi, Paolo Sabbatani, Stefano Severi, Cristiana Corsi

**Affiliations:** ^1^Computational Physiopathology Unit, Department of Electrical, Electronic and Information Engineering “Guglielmo Marconi”, University of Bologna, Bologna, Italy; ^2^Electrophysiology Laboratory, Cardiology Unit, Ravenna and Cesena Hospitals, Azienda Unità Sanitaria Locale della Romagna, Ravenna, Italy

**Keywords:** atrial fibrillation, cathether ablation, rotor detection, computational modeling, synthetic electrograms, mapping catheter geometry

## Abstract

Atrial fibrillation (AF) is the most common cardiac arrhythmia and catheter mapping has been proved to be an effective approach for detecting AF drivers to be targeted by ablation. Among drivers, the so-called rotors have gained the most attention: their identification and spatial location could help to understand which patient-specific mechanisms are acting, and thus to guide the ablation execution. Since rotor detection by multi-electrode catheters may be influenced by several structural parameters including inter-electrode spacing, catheter coverage, and endocardium-catheter distance, in this study we proposed a tool for testing the ability of different catheter shapes to detect rotors in different conditions. An approach based on the solution of the monodomain equations coupled with a modified Courtemanche ionic atrial model, that considers an electrical remodeling, was applied to simulate spiral wave dynamics on a 2D model for 7.75 s. The developed framework allowed the acquisition of unipolar signals at 2 KHz. Two high-density multipolar catheters were simulated (Advisor™ HD Grid and PentaRay^®^) and placed in a 2D region in which the simulated spiral wave persists longer. The configuration of the catheters was then modified by changing the number of electrodes, inter-electrodes distance, position, and atrial-wall distance for assessing how they would affect the rotor detection. In contact with the wall and at 1 mm distance from it, all the configurations detected the rotor correctly, irrespective of geometry, coverage, and inter-electrode distance. In the HDGrid-like geometry, the increase of the inter-electrode distance from 3 to 6 mm caused rotor detection failure at 2 mm distance from the LA wall. In the PentaRay-like configuration, regardless of inter-electrode distance, rotor detection failed at 3 mm endocardium-catheter distance. The asymmetry of this catheter resulted in rotation-dependent rotor detection. To conclude, the computational framework we developed is based on realistic catheter shapes designed with parameter configurations which resemble clinical settings. Results showed it is well suited to investigate how mapping catheter geometry and location affect AF driver detection, therefore it is a reliable tool to design and test new mapping catheters.

## Introduction

Mechanisms responsible for atrial fibrillation (AF) initiation and maintenance are still largely debated, and different hypotheses have been formulated. While the ectopic activity arising primarily from veins and veno-atrial junctions has been accepted as the main initiating mechanism in paroxysmal AF, arrhythmia perpetuation remains a widely discussed issue. Two main hypotheses so far prevail: local drivers and multiwavelet re-entries. The former proposes a stationary driver generating waves that propagate to the atria passively and in a non-uniform way, constituting “fibrillatory conduction.” The latter hypothesis is related to self-sustaining, moving functional reentrant circuits without any stable driver. Several combinations of such mechanisms from drivers to multiwavelets in time have been subsequently proposed. The “rotors theory,” proposed by Narayan et al. (2012), hypothesized that some fairly stable electrical rotors can be traced and targeted on a patient-specific-based approach for AF ablation.

Transcatheter ablation is a widely accepted therapeutic option to try to eliminate atrial mechanisms responsible for the genesis and maintenance of the arrhythmia. Several energy sources have been used, applied either one-shot or point-to-point: in the latter, notably with radiofrequency energy delivery, catheter mapping is usually performed to investigate mechanisms that trigger and sustain AF (Kusumoto et al., 2018; Cheung et al., 2021). Intracardiac mapping catheters allow an accurate anatomical reconstruction and endocardial voltage mapping; this information underpins the effective ablation of the arrhythmia. For an exhaustive and comprehensive review of cardiac mapping readers should refer to Shenasa et al. (2019).

Beyond the controversies still surrounding the “rotors theory,” (Allessie and de Groot, 2014a,b; Narayan and Jalife, 2014), ablation of rotors suffers from a major technological issue for tracking real-time atrial electrical activities since different multi-electrode/high-density electrophysiological mapping catheters can lead to different reconstructions. In this scenario, in the last years, computational AF mapping methods have been developed to provide a patient-specific analysis of wave propagation to identify the rotor tip position. Several studies have assessed the technical capabilities and limitations of mapping catheters used in clinical practice to detect such targets (Allessie and de Groot, 2014b; Roney et al., 2017b). However, the development of reliable and accurate tools for locating such sources remains a major challenge. Since rotor detection by multi-electrode catheters may be influenced by several parameters including inter-electrode spacing, catheter coverage, and endocardium-catheter distance, in this work: (i) we used computer simulations to overcome clinical limitations in studying these factors influencing the precision of multi-electrode mapping; and (ii) we developed a tool which allows the testing of different catheters shapes in different conditions and their behavior in detecting a synthetic rotor.

## Materials and Methods

### Cardiac Tissue Simulation

In our model we considered the atrial tissue as a two-dimensional grid of cells of size 5 × 5 cm with a distance between cells of 0.25 mm, using the monodomain approach with a fixed time step of 10μs, Neumann boundary conditions, and a modified Courtemanche et al. (1998). action potential (AP) model which accounts for AF related ionic remodeling. The AF condition was simulated by modification of selected sarcolemmal ion channel conductivities: I_to_ and I_CaL_ were each decreased by 65%, I_Kur_ was decreased by 49% and I_K1_ increased by 110% (Wilhelms et al., 2013). The electrical propagation of the atrial AP was modeled by the following monodomain reaction-diffusion equation:


(1) $$\frac{\partial V_{m}}{\partial t}=D_{x}\,\frac{\partial^{2}V_{m}}{\partial^{2}x}+D_{y}\,\frac{\partial^{2}V_{m}}{\partial^{2}y}-\frac{I_{i\,o\,n}}{C_{m}}-\frac{I_{s\,t\,i\,m}}{C_{m}}$$

where *D* = [*D*_*x*_ 0;0*D*_*y*_] is the conductivity tensor with *D*_*x*_ = *D*_*y*_ = 0.031*mm*^2^/*ms*as in Bai et al. (2019), *I*_*ion*_ is the total ionic current that crosses the membrane cells, *V_m_* is the membrane potential, *I*_*stim*_ is the stimulus current, and the membrane capacitance *C_m_=100pF* as in Courtemanche et al. (1998). The plane wave conduction velocity (CV) we obtained was 26 cm/s. The numerical integration for the whole system (monodomain and ionic model) was made using the forward Euler scheme and solved with Matlab. The system was discretized in space using second-order centered finite differences. For triggering the spiral wave, we applied both a stimulus (stimulus current amplitude –2000 pA with duration = 1 ms) at *t* = 0 to the bottom line of the grid and an extrastimulus of the same amplitude at *t* = 105 ms (with duration = 0.25 ms) on the left bottom corner (25 × 25 cells) of the simulated tissue. In the monodomain, the phase map was computed, and the position of the true phase singularity (PS) was detected through the whole simulation time by applying the model proposed by Zhuchkova and Clayton (2005) (Supplementary Video 1).

### Electrogram Computation and Catheters Configuration

**FIGURE 1:**
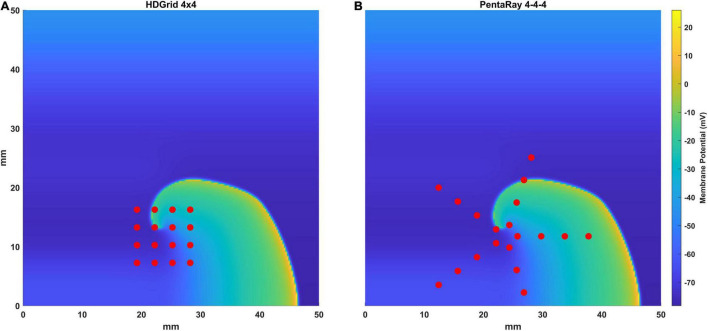
Representation of the simulated catheters [the HD Grid **(A)** and the PentaRay **(B)**] superimposed to the simulated electrical propagation on the atrial tissue in one-time frame. Each electrode is represented with a red dot.

We simulated two of the most used mapping catheters in clinical electrophysiology: the Advisor™ HD Grid Mapping catheter (grid-shaped, 16 electrodes, evenly spaced 3 mm apart, from now on HDGrid), and the PentaRay^®^ NAV catheter (star-shaped, 5 splines with 4 electrodes each, evenly spaced along the spline 4 mm apart, from now on PentaRay) (Figure 1).

The catheters were initially placed on the atrial wall tissue in such a way that the centroid of the electrodes’ position corresponded to the average position of the true phase singularity from the monodomain tissue simulation, throughout the simulation time. Thereafter, two different shifted conditions were tested in relation to the ground truth rotor tip position: for the 4 × 4, 3 mm inter-electrode distance HDGrid, the shift to the right was 1.5 and 3 mm; for the 4-4-4 PentaRay it was of 5 and 8 mm.

To test the performance of the catheters to correctly detect arrhythmia drivers, we considered the following factors: (i) the electrode-wall distance, (ii) the inter-electrode distance, and (iii) the coverage.

For both catheters, different positions were tested in terms of distance from the LA wall. In particular, the catheter’s electrodes were positioned at a set of constant distances *z_0_* from the atrial tissue; in the case of electrodes in contact with the atrial wall a minimum distance *z_0_* of 0.25 mm, corresponding to the distance between cells in the grid, was used. Then, to test the effect of a non-perfect contact between the catheters and the atrial wall, for both catheters, their distance from the atrial wall was increased to 1, 2, and 3 mm. Moreover, some illustrative conditions in which different electrodes are at different distances were simulated. For the HDGrid three conditions were analyzed: one considering the two external splines at 1 mm from the tissue and the central splines in contact; the second with the external splines in contact and the central splines at 1 mm; and the third with the two left splines in contact and the other two at increasing distances (2 and 3 mm, respectively). For the PentaRay two conditions were tested: the five electrodes of the central ring at 1 mm and the two central rings at 2 and 3 mm distance.

For the HDGrid, we considered 2 configurations to simulate different coverages: 16 electrodes in a 4 × 4 layout and 36 electrodes in a 6 × 6 layout.

In addition, the inter-electrode distance was also changed in the range of 3–6 mm. Similar to the HDGrid, the PentaRay was simulated in two configurations: in each of the five splines, the four electrodes were placed at 4-4-4 and 2-2-2 mm distances.

For each electrode, the electrogram was obtained as a weighted summation of the effects of every single cell considered as a dipole and a sampling frequency of 1 kHz (Spach et al., 1979; Shillieto et al., 2016).


(2) $$e\,g\,m_{u\,n\,i\,p\,o\,l\,a\,r}\propto \sum_{i}\frac{\left(\partial_{x\,x}V_{m_{i}}+\partial_{y\,y}V_{m_{i}}\right)}{\sqrt{d_{i}^{2}+z_{0}^{2}}}$$

in which *∂*_*xx*_⁡*V_mi_* and *∂*_*yy*_⁡*V_mi_* are the second derivatives along the *x* and *y* axis of the transmembrane potential *V*_*m*_(*x*,*y*,*t*) of the i-th cell and *d*_*i*_ is the distance of the electrode from each cell. For this purpose, we assumed that the electrograms were recorded with point electrodes whose diameters need not be considered considering the size of the catheters and the amplitude of the rotor tip trajectory (Abdi et al., 2021). White Gaussian noise was added to each synthesized EGMs with a signal-to-noise ratio of 50 dB.

**FIGURE 2:**
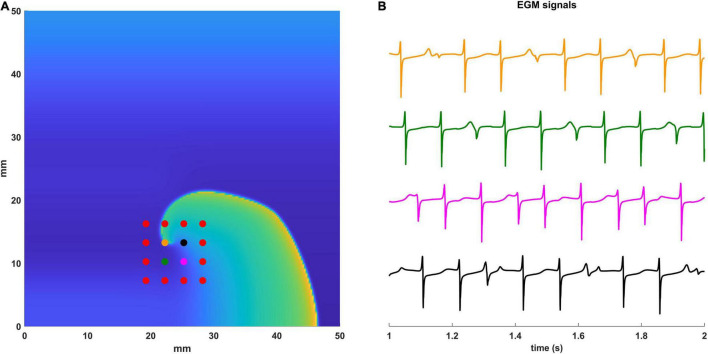
Simulated EGM signals. **(A)** Computational configuration with the 16 electrodes of the 3 mm HD Grid catheter. Electrodes for which the EGM signals are displayed are in different colors. **(B)** The simulated EGM signals are shown between 1 and 2 s of simulation. Electrodes and the corresponding EGMs are coded with the same color.

In Figure 2 we show an example of the computational configuration of the 16 electrodes of the 3 mm HDGrid catheter (panel A) and the EGM signals (panel B) acquired by the four central electrodes.

In addition to the previous analysis, we also tested the proposed workflow on a real anatomical atrial shape acquired during ablation in a patient affected by AF. The cardiac tissue simulation was projected on a real atrial anatomy and the catheter was located in contact with the LA wall to acquire EGMs.

### Catheter-Based Phase Mapping

Spatiotemporal organization of atrial fibrillation was studied applying a previously developed and validated algorithm (Valinoti et al., 2017) based on phase analysis for local atrial activation timings (LATs) detection and on the persistence of phase singularities for meandering and stable rotor identification on the left atrium (LA) wall.

The EGM signals first were filtered with a band-pass filter (3–80 Hz). The algorithm for LAT detection was designed as a modified version of the electrogram recomposition from sinusoidal wavelets proposed by Kuklik et al. (2015) and Valinoti et al. (2017, 2018). In the proposed sinusoidal recomposition algorithm, the wavelets were only generated in correspondence of a typical morphology of atrial activations; the additional constraints to be satisfied for sinusoidal wavelet generation took into consideration the slope of negative deflections, their amplitude, and duration. The resulting phase inversion points were then used to center a window of fixed duration in which the point with the maximum negative derivative corresponded to the LATs. Once the LATs were detected, 2D phase maps were reconstructed, in the portion of tissue under coverage by the catheter, by assigning to each point the phase value from the nearest electrode, up to a certain maximum distance (cut_distance: 3 mm for HD Grid and 12 mm for PentaRay). Phase singularities were then defined as a point whose neighboring region is characterized by a gradual phase transition followed by an abrupt phase inversion (from π to –π). Since we have a discrete geometry, where every electrode is a point, we search for the smallest closed-loop, with the aforementioned described properties. If the closed-loop satisfies those properties a phase singularity is placed in the midpoint of the electrodes involved.

### Rotor Tracking

At any given time sample the algorithm might detect more than one PS point, due to discretization of the electrodes. At the end of this step for each time sample, we may have zero, one, or more PSs. Starting from the last time sample, and going backward, the PSs are linked to the previous ones based on their temporal and spatial distance. Two parameters are defined, a temporal one that is the maximum gap to be filled when no PS is detected, and a spatial one that is the fixed maximum distance from the PS in the current frame within which to track backward the closest PS in the neighboring regions. For this reason, the adequate temporal and spatial conditions must be met for a series of phase singularities to be detected as part of a rotor. Eventually, only the phase singularities which persist for more than two dominant periods are defined as rotors.

### Data Visualization With Density Maps

For a visual representation of the phase singularities belonging to the rotor, a phase singularities density map was constructed. Considering only the longest rotor, for each detected PS belonging to it, the centroid of the electrodes which contain the smallest closed-loop was used to place a bidimensional Gaussian distribution, of standard deviation equal to half the inter-electrode distance in the HD Grid.

The phase singularity density was the function of the position (*x*,*y*), with the following formula (Alessandrini et al., 2018):


(3) $$P\,S\,d\,\left(x,y\right)=\frac{1}{2\,\pi \,\sigma^{2}}\,\sum_{i=1}^{N}e^{-\frac{{\left(x-x_{P\,S\,i}\right)}^{2}+{\left(y-y_{P\,S\,i}\right)}^{2}}{2\,\sigma^{2}}}$$

*x*_*PSi*_ and *y*_*PSi*_ were the coordinates of the i-th PS, and σ set to 1.5 mm as specified before.

The contributions from all the phase singularity were summed up on a grid with a resolution of 0.25 mm for visualization.

### Hardware and Software

The monodomain simulation and the rotor tracking algorithm were implemented in MATLAB (release R2019b) and run on a workstation equipped with 64 GB of RAM and a ThreadRipper 3990 × CPU. Computation time for the simulation of a catheter configuration was of 15 min each. Density maps visualization from the data was instantaneous, while the rendering of the activation map for the whole simulation required half an hour. The software will be available upon request to the authors.

## Results

### Simulation of HDGrid Catheters: Effect of Electrode-Wall Distance

This simulation was carried out considering the 4 × 4 layout with a 3 mm inter-electrode distance. We computed the electrograms and the corresponding phase maps with the catheter in contact with the LA wall (0 mm) and at 1, 2, and 3 mm. The HDGrid was positioned to cover the ground truth rotor trajectory as much as possible, and then moved far from the wall to test the robustness of the catheter configuration in detecting rotors in non-ideal contact conditions as well.

**FIGURE 3:**
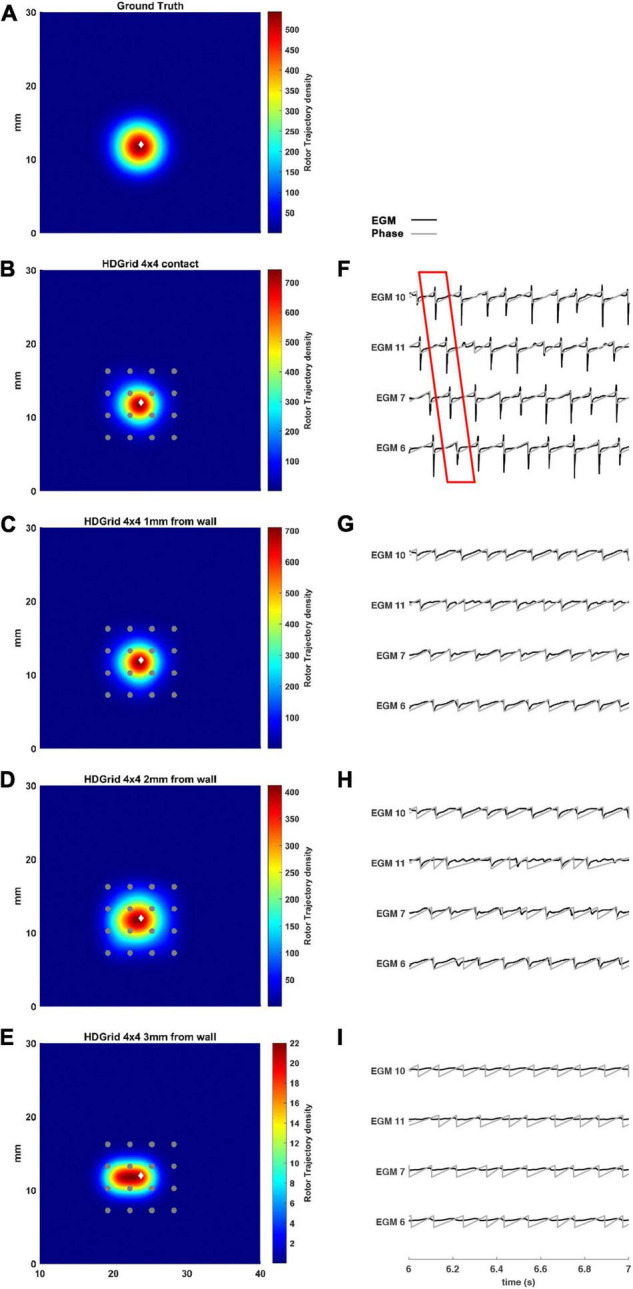
Density maps of rotor tip trajectory reconstructed with an inter-electrode distance of 3 mm, with a 4 × 4 HD Grid catheter at different distances from the atrial wall. **(A)** Ground truth; **(B)** 3 mm HD Grid catheter in contact; **(C)** 3 mm HD Grid catheter at 1 mm from the wall; **(D)** 3 mm HD Grid catheter at 2 mm from the wall; **(E)** 3 mm HD Grid catheter at 3 mm from the wall. The patch has been zoomed (x-axis between 10 and 40 mm, y-axis between 0 and 30 mm). Color encodes the trajectory density value. **(F–I)** The phase activation (gray line) and EGM signal (black line) for the 4 central electrodes (n. 6-7-10-11) are reported under each simulated configuration at the same time frame. The white diamond marker represents the rotor tip position of the ground truth.

The estimated rotor tip density maps in these four different conditions are shown in Figure 3. It can be observed that in the contact condition the algorithm detects the rotor with a perfect agreement with ground truth (Figures 3A,B): the peak-to-peak distance is 0 mm and the duration of the detected rotor is almost equal to the ground truth (7.71 s vs. 7.75 s, Table 1). By varying the distance from the atrial wall (Figures 3C–E), it was possible to locate the rotors correctly up to a distance of 2 mm, whereas, at 3 mm, the phase singularities were lost; subplots in Figure 3F–I show how the EGMs have been deformed and the activation phase lost. This comparison can be also appreciated in the Supplementary Video 2, showing the rotor tracking in the contact (left) condition and at 3 mm wall distance (right).

**TABLE 1:** Simulation parameters and resulting indexes derived from the phase singularity maps for ground truth, HDGrid and PentaRay catheters, varying coverage (number and spacing between electrodes), and wall distance.

	Inter-electrode distance (mm)	Distance from the atrial wall (mm)	Rotor duration (s)	Density peak	Standard deviation (mm)	Peak to peak distance between ground truth and estimated rotor (mm)
Ground truth			7.75	544	8.23	
HD Grid 4 × 4	3	0	7.71	743	1.78	0
		1	7.74	711	1.88	0
		2	6.7	413	2.54	0.25
		3	0.25	22	1.47	1
	6	0	7.7	1078	0.6	0.25
		1	7.68	1052	1.41	0.25
		2	1.36	154	2.16	5.75
		3	0.28	18	4.29	6
HD Grid 6 × 6	3	0	7.71	741	1.8	0
PentaRay	4-4-4	0	7.71	884	1	0.25
		1	7.69	841	1.26	0.25
		2	7.72	288	4.7	1.27
		3	7.64	281	6.25	9.5
	2-2-2	0	7.72	963	0.76	0.25
		1	7.71	945	0.81	0.25
		2	7.73	440	2.8	0.79
		3	5.3	261	2.96	1.35

### Simulation of HDGrid Catheters: Effect of Inter-Electrode Distance and Coverage

**FIGURE 4:**
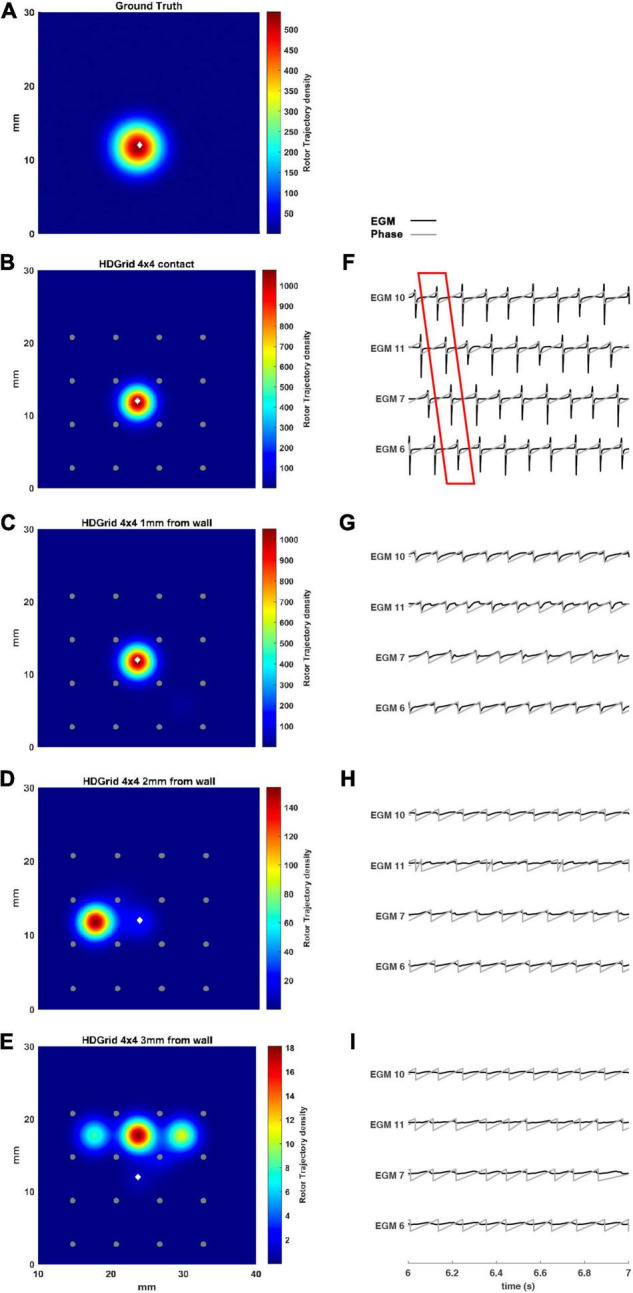
Density maps of rotor tip trajectory reconstructed with an inter-electrode distance of 6 mm, with a 4 × 4 HD Grid catheter at different distances from the atrial wall. **(A)** Ground truth; **(B)** 6 mm HD Grid catheter in contact; **(C)** 6 mm HD Grid catheter at 1 mm from the wall; **(D)** 6 mm HD Grid catheter at 2 mm from the wall; **(E)** 6 mm HD Grid catheter at 3 mm from the wall. The patch has been zoomed (x-axis between 10 and 40 mm, y-axis between 0 and 30 mm). Color encodes the trajectory density value. **(F–I)** The phase activation (gray line) and EGM signal (black line) for the 4 central electrodes (n. 6-7-10-11) are reported under each simulated configuration at the same time frame. The white diamond marker represents the rotor tip position of the ground truth.

The present tool also considered the effect of inter-electrode distance and coverage on the rotor detection. For this reason, the inter-electrode distance was varied between 3 (default) and 6 mm. In our simulation (Figures 4B,C,F,G) for the contact configuration and 1 mm of distance wall, the rotor was still correctly detected compared to the ground truth (Figure 4A). The combined effects of increasing inter-electrode distance and the distance from the wall were also analyzed. In this configuration at 2 and 3 mm from the LA wall (Figures 4D,E), the driver was lost: the EGM signals and the derived activation phase are deformed (Figures 4H,I). By increasing the inter-electrode distance from 3 to 6 mm the precise location of the driver was lost at a smaller catheter-wall distance (2 mm vs. 3 mm). Moreover, the driver position was not accurately estimated (compare Figure 4D vs. Figure 3E).

**FIGURE 5:**
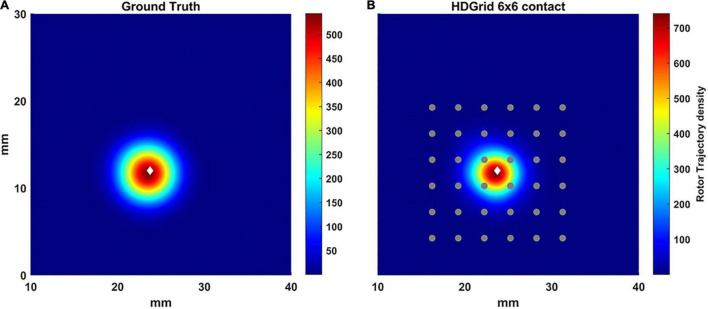
Density maps of rotor tip trajectory reconstructed by the algorithm with an inter-electrode distance of 3 mm with a 6 × 6 layout of the HD Grid catheter. **(A)** Ground truth; **(B)** 3 mm 6 × 6 HD Grid catheter in contact. The patch has been zoomed (x-axis between 10 and 40 mm, y-axis between 0 and 30 mm). Color encodes the trajectory density value. The white diamond marker represents the rotor tip position of the ground truth.

To vary the coverage, we also increased the number of the electrodes by simulating a 6 × 6 HDGrid configuration (Figure 5), 3 mm inter-electrode distance. The density map and the features in Table 1 confirmed the ability of the algorithm for rotor detection, in line with the 4 × 4 HDGrid results.

### Simulation of PentaRay Catheters: Effect of Electrode-Wall Distance

**FIGURE 6:**
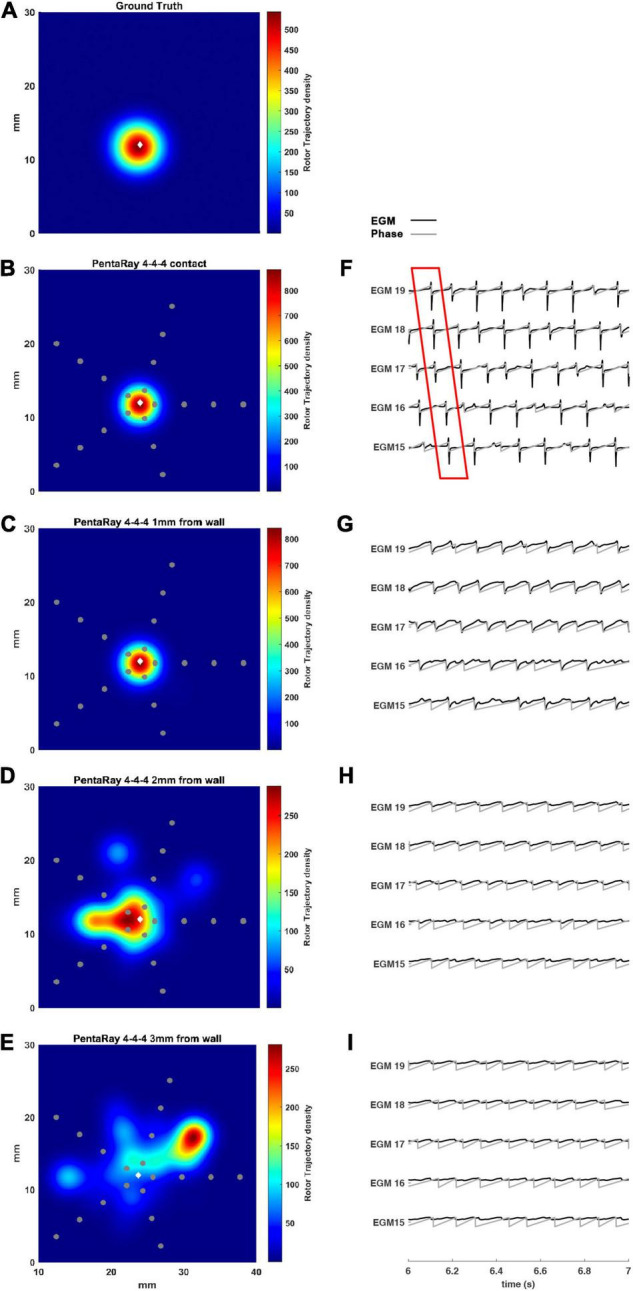
Density maps of rotor tip trajectory reconstructed by the 4-4-4 PentaRay catheter at different distances from the atrial wall. **(A)** Ground truth; **(B)** PentaRay catheter in contact; **(C)** PentaRay catheter at 1 mm from the wall; **(D)** PentaRay catheter at 2 mm from the wall; **(E)** PentaRay catheter at 3 mm from the wall. The patch has been zoomed (x-axis between 10 and 40 mm, y-axis between 0 and 30 mm). Color encodes the trajectory density value. **(F–I)** The phase activation (gray line) and EGM signal (black line) for the central electrodes (n. 15-16-17-18-19) are reported under each simulated configuration at the same time frame. The white diamond marker represents the rotor tip position of the ground truth.

In Figure 6, we simulated a 4-4-4 PentaRay in contact with the LA wall (Figures 6B–F) and at 1, 2, and 3 mm from the LA wall (Figures 6C–I, respectively). As for the HDGrid at 3 mm of distance, the detection of the rotors was missed; the peak of density was strongly reduced and the peak to peak distance between ground truth and estimated rotor increased. Figures 6F–I show the modified ECG and phase activation for the 5 central electrodes of the PentaRay catheter: the signals started to get worse as he wall distance increased. Rotor tracking with the PentaRay catheter is also shown in the Supplementary Video 3.

### Simulation of PentaRay Catheters: Effect of Coverage

**FIGURE 7:**
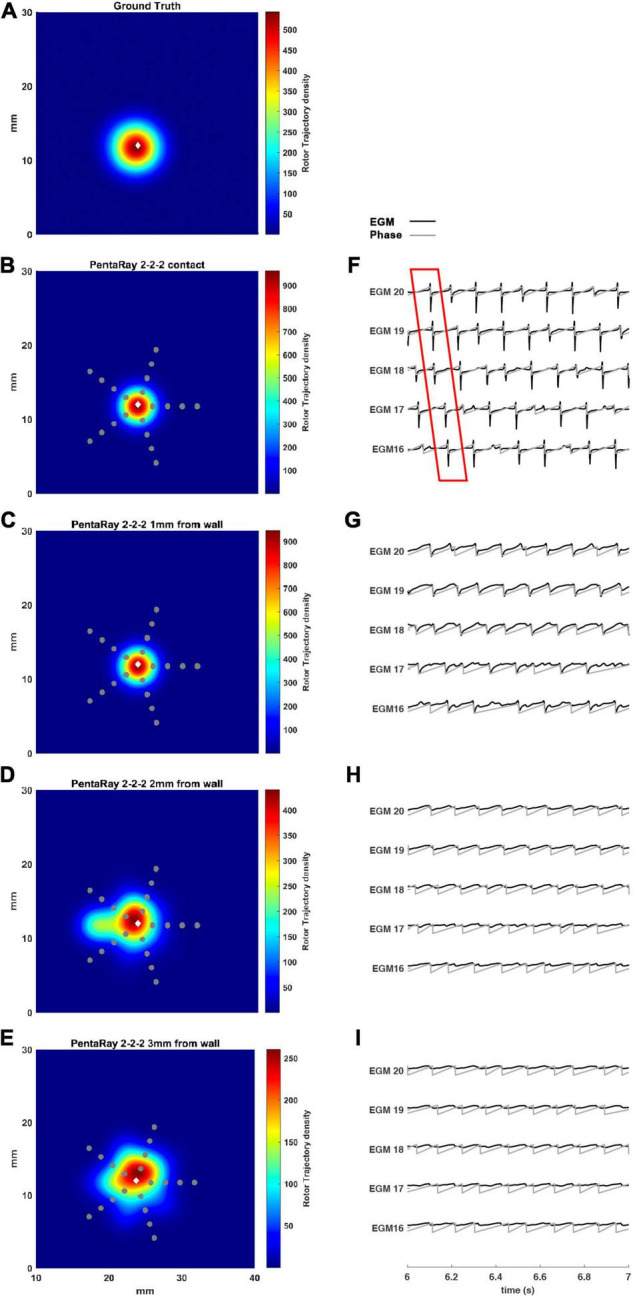
Density maps of rotor tip trajectory reconstructed by the 2-2-2 PentaRay catheter at different distances from the atrial wall. **(A)** Ground truth; **(B)** PentaRay catheter in contact; **(C)** PentaRay catheter at 1 mm from the LA wall; **(D)** PentaRay catheter at 2 mm from the LA wall; **(E)** PentaRay catheter at 3 mm from the LA wall. The patch has been zoomed (x-axis between 10 and 40 mm, y-axis between 0 and 30 mm). Color encodes the trajectory density value. **(F–I)** The phase activation (gray line) and EGM signal (black line) for the central electrodes (n. 16-17-18-19-20) are reported under each simulated configuration at the same time frame. The white diamond marker represents the rotor tip position of the ground truth.

We tested the 2-2-2 PentaRay configuration to evaluate the coverage effects (Figure 7). In this case, there were no significant differences compared to the 4-4-4 PentaRay configuration when the catheter was in contact with the LA wall; as expected, the peak of the density map increased but the position of the driver was correctly identified. However, when the distance from the LA wall was increased, the high resolution of the catheter allowed the detection of the rotor for a distance greater than 2 mm as well.

The asymmetric configuration of the PentaRay catheter suggested that an additional test be made to check if the specific orientation of the splines, which could make them more or less superimposed to the rotor tip trajectory, affects the detection results. To this aim, we considered the rotation of the catheter by half of the angle between two splines (36°), which makes the splines’ orientations more different from the starting one. We simulated such configuration for both the 2-2-2 and 4-4-4 PentaRay at 2 mm distance from the LA wall. Results are reported in Table 2, showing a large improvement in terms of peak to peak distance between ground truth and estimated rotor after rotating the catheter.

**TABLE 2:** Results obtained by rotating the PentaRay catheter by 36°.

	Inter-electrode distance (mm)	Distance from the atrial wall (mm)	Rotor duration (s)	Density peak	Standard deviation (mm)	Peak to peak distance between ground truth and estimated rotor (mm)
Ground truth			7.75	544	8.23	
PentaRay	4-4-4	2	7.72	288	4.57	1.27
	2-2-2	2	7.72	440	2.8	0.79
Rotated	4-4-4	2	7.72	433	3.8	0
PentaRay	2-2-2	2	7.72	547	2.33	0.5

### Effects of the Relative Position of the Catheter With Respect to the Rotor Tip

**FIGURE 8:**
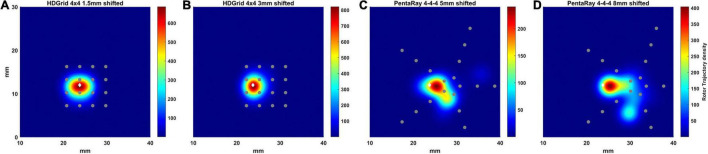
Density maps of rotor tip trajectory reconstructed in the shifted tests. **(A)** 4 × 4 HD Grid catheter with an inter-electrode distance of 3 mm and a positive shift of 1.5 mm on the right; **(B)** 4 × 4 HD Grid catheter with an inter-electrode distance of 3 mm and a positive shift of 3 mm on the right; **(C)** 4-4-4 PentaRay catheter with a right shift of 5 mm; **(D)** 4-4-4 PentaRay catheter with a right shift of 8 mm. The patch has been zoomed (x-axis between 10 and 40 mm, y-axis between 0 and 30 mm). Color encodes the trajectory density value. The white diamond marker represents the rotor tip position of the ground truth.

To simulate real conditions, in which the spiral tip position is not known *a priori*, we added some simulations in which the rotor tip was not located in the middle of the catheters. We tested a shift of the catheter position in relation to the default rotor tip central position for both catheters’, still allowing coverage of the rotor meandering, since we aimed to assess if the catheter design affects the rotor detection (see Figure 8).

In the case of HDGrid, a shift of 1.5 and 3 mm (Figures 8A,B, respectively) did not significantly affect the capability of locating the rotor tip position based on the peak of the estimated density map (Table 3). It might be unexpected that the estimated density map of rotor trajectory was more scattered for the shorter shift (compare Figure 8A with Figure 8B), however this was a clear consequence of the discretized mapping available through the catheter: when only four electrodes were involved in the phase inversion due to the presence of the rotor its position was unequivocally estimated in the centroid of those four electrodes (Figure 8B). On the other hand, when the rotor tip meandered and six different electrodes were involved (depending on time) the density map is horizontally spread (as in Figure 8A). Overall, the highly symmetric distribution of the electrodes within the catheter made the estimates very stable up to where the rotor was located within the (quite limited) covered area. To enforce this result, we performed a simulation in which we sequentially moved the HDGrid catheter for covering the entire lower part of the domain (Supplementary Figure 1). False rotors were not detected, and, even without *a priori* knowledge of the real rotor position, the catheter assessed its location correctly.

**TABLE 3:** Simulation parameters and resulting indexes derived from the phase singularity maps for ground truth, HDGrid, and PentaRay catheters with the shift and no-contact electrodes simulations.

	Shift x (mm) (see Figure 8)	Distance from the atrial wall (mm)	Rotor duration (s)	Density peak	Standard deviation (mm)	Peak to peak distance between ground truth and estimated rotor (mm)
Ground Truth			7.75	544	8.23	
HDGrid 4 × 4	1.5	0	7.72	686	1.64	0.25
	3	0	7.71	821	1.56	0.25
PentaRay	5	0	3.53	241	3.2	1.75
4-4-4	8	0	6.7	404	3.6	2
	**No contact electrodes (see Figure 9)**					
HDGrid 4 × 4	2 central splines	1	7.72	439	2.69	0.25
	2 lateral splines	1	7.71	790	1.56	0
	3rd/4th splines	2/3	6.49	620	1.85	0.56
PentaRay	central ring	1	7.7	567	1.96	0.35
4-4-4	two central rings	2/3	5.47	176	4.18	2.66

In the case of PentaRay when the rotor tip was located peripherally in relation to the catheter center (Figures 8B,C) its estimated position was more significantly, though not dramatically, affected. In particular, shifts of 5 and 8 mm led to errors of 1.75 and 2 mm in the estimate, respectively. In the latter case, the less robust estimation of rotor dynamics was also witnessed by the reduced estimated time duration of the rotor (Table 3).

### Effects of Various Distances of the Electrodes From the Atrial Wall

**FIGURE 9:**
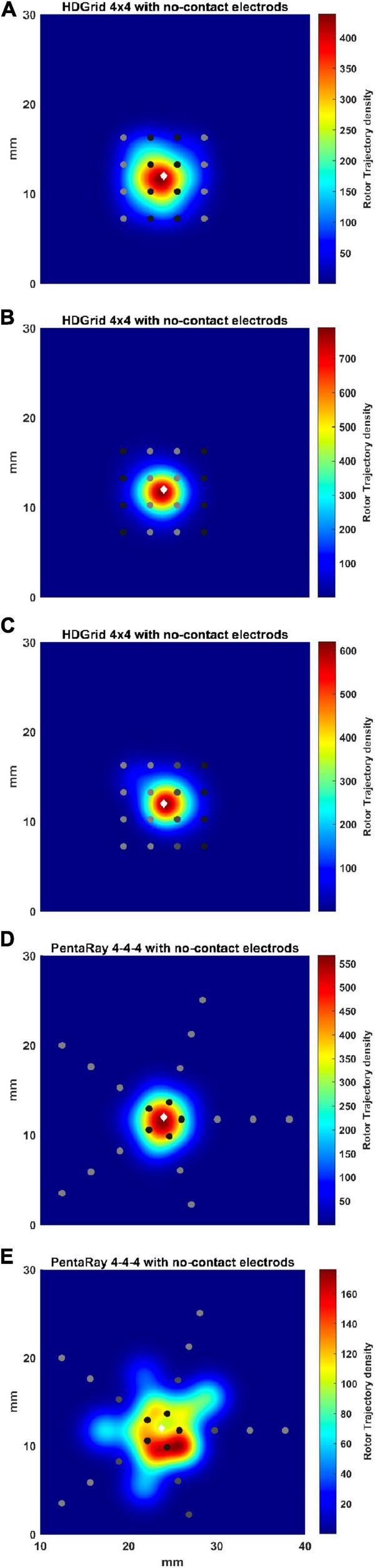
Density maps of rotor tip trajectory reconstructed in the no-contact electrodes conditions. **(A)** 4 × 4 HD Grid catheter with an inter-electrode distance of 3 mm: the external splines are in contact (light gray) while the central splines are at a distance of 1 mm from the wall (dark gray color); **(B)** 4 × 4 HD Grid catheter with an inter-electrode distance of 3 mm: the central splines are in contact (light gray) while the external splines are at a distance of 1 mm from the wall (dark gray); **(C)** 4 × 4 HD Grid catheter with an inter-electrode distance of 3 mm: the two left splines are in contact (light gray) while the other two splines are at increase distance of 2 (dark gray) and 3 mm from the wall (dark), respectively; **(D)** 4-4-4 PentaRay catheter: the central ring is at a distance of 1 mm from the wall (dark gray); **(E)** 4-4-4 PentaRay catheter: the two central rings are at increase distance of 2 mm (dark gray) and 3 mm from the wall (dark), respectively. The patch has been zoomed (x-axis between 10 and 40 mm, y-axis between 0 and 30 mm). Color encodes the trajectory density value. The white diamond marker represents the rotor tip position of the ground truth.

During data acquisition, some electrodes may be in contact while others may not. We performed some illustrative simulations by considering different cases in which different electrodes were at different distances (Figure 9 and Table 3). In particular, simulations in which only some splines of the catheter are in contact with the simulated tissue can be assumed as an approximated way to simulate 3D complex geometries in which the concavity of the LA surface may not allow a perfect contact between the mapping catheter and the endocardial wall.

For the HDGrid in all three simulated conditions, in which only some splines were in contact (Figures 9A–C), the estimation proved to be very robust, with only minor variations (Table 3).

For the PentaRay, having only the central ring not in contact did not affect the estimate (Figure 9D), whereas the progressive detachment of two internal rings (Figure 9E) substantially worsened the estimation results (Table 3).

### Results on a Real Anatomy of an Atrial Fibrillation Patient

**FIGURE 10:**
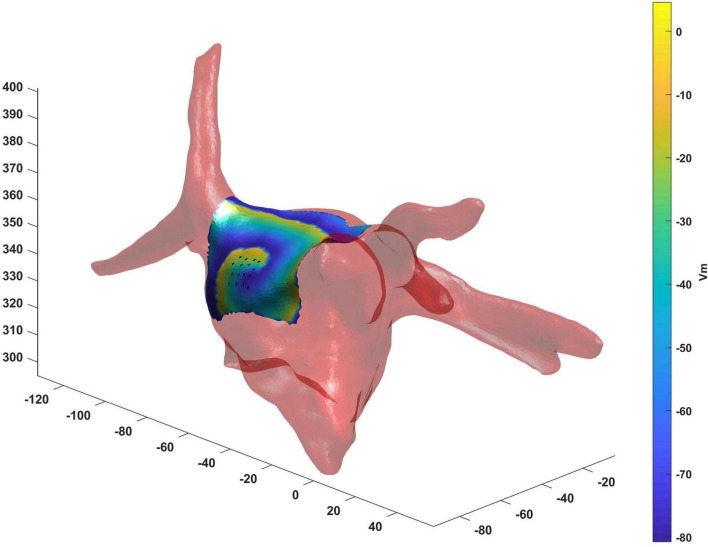
Real atrial shape (light red) from a patient acquired during ablation on which we have tested the proposed framework. The 3D region covered by the catheter (black points) is represented by the tissue in which the voltage was simulated. Our framework was able to detect correctly the rotor tip (duration of the detected rotor 7.75 s, mean distance between ground truth and detected rotor tip 2 mm).

The real atrial shape acquired during ablation on a patient affected by AF is shown in Figure 10. The 3D region covered by the catheter is represented by the tissue in which the voltage was simulated. Our framework was able to detect correctly the rotor for a total duration of 7.75 s. The mean distance between ground truth and detected rotor tip was 2 mm.

## Discussion

Our work proposed a new tool to test the geometric configurations of two commercial mapping catheters, the HDGrid and the PentaRay, in different conditions, and their ability to detect synthetic rotors.

Our results showed that the proposed tool allowed the simulation of both commercial high-resolution catheters and catheters in which parameters such as the number of electrodes, inter-electrode distance, and endocardium-catheter distance were changed. In particular, when the distance between the catheter and cardiac tissue was set to 0 (in contact with the endocardial wall) all the configurations detected the rotor correctly, irrespective of geometry, coverage, and inter-electrode distance. The most critical parameter for rotor detection was in fact electrodes’ distance from the cardiac wall. In the HD Grid-like geometry, at a wall distance of 3 mm, the electrograms became too smoothed (Figures 3I–4I) to consistently detect the activation, and phase singularities were lost. Decreasing the catheter resolution (inter-electrode distance from 3 to 6 mm) impeded rotor detection at a closer distance (2 mm) from the LA wall. In the PentaRay-like configuration, at higher resolution (inter-electrode distance equals to 2-2-2 mm) the rotor detection only mildly worsened at 3 mm endocardium-catheter distance whereas it failed at lower resolution (4-4-4 mm) (Table 1 and Figures 6I–7I). Notably, the asymmetry of such a catheter resulted in rotation-dependence of the rotor detection, which is consistent with the fact that, depending on the orientation of the splines, the rotor tip in its meandering keeps closer to them or covers the inter-spline regions, where the distance from electrodes increases.

Our main finding is that the inter-electrode distance is critical for rotor detection when the distance between the catheter and LA wall increases for both the tested catheter shapes. The higher resolution in the central part of the catheter also seems to underlie the better performance of the PentaRay 2-2-2 compared with the HD Grid 4 × 4 at 3 mm from LA wall. Moreover, both the PentaRay 4-4-4 and the HD Grid 4 × 4, at both tested interelectrode distances (3 and 6 mm), lose the correct detection by increasing catheter-LA distance.

In the last decades, several works have analyzed atrial EGMs acquisition by different catheters using a simulation approach (Alessandrini et al., 2017; Roney et al., 2017b; Martinez-Mateu et al., 2018, 2019; Hwang et al., 2019; Ravikumar et al., 2021), or have developed methods for rotor tracking with simulated and/or experimental EGMs (Roney et al., 2014; Ugarte et al., 2015; Valinoti et al., 2015, 2017; Ganesan et al., 2019a,b).

Only a few studies (Roney et al., 2017a; Martinez-Mateu et al., 2018, 2019) have dealt directly with the effects of inter-electrode distance, coverage, and distance from the atrial wall and the comparison of different commercial catheters for rotor detection.

In Roney et al. (2017a) defined the minimum spatial resolution requirements, as a function of AF wavelength (WL), to correctly identify the AF driver. They tested three simulated high-resolution catheters (Lasso, AFocus, PentaRay) placed over the rotor core. They found that the minimum spatial resolution was WL/3.1. In our simulations, WL was about 20 mm, which would give a minimum required resolution of 6.5 mm. Indeed, at contact, all the tested resolutions up to 6 mm were effective in locating the rotor. Importantly, our results suggest that the concept of a minimum resolution requirement should be considered cautiously in a real-life scenario, since we have clearly demonstrated that, if the catheter is not in contact, the performance rapidly worsens compared with this ideal case. Compared to Roney et al. (2017a), in our study we tested another catheter whose use nowadays is widespread, the HDGrid catheter, showing an impressive performance also when inter-electrode distance almost equals the minimum spatial resolution (6 mm). Moreover, the performance of each catheter was tested when it was not placed over the rotor core (Figure 8), and considering some non-contact electrodes (Figure 9); the PentaRay configuration was particularly sensitive to the former, whilst the detection through the HDGrid was robust in both conditions. This information provides additional insights into catheter performance in a real-life scenario.

The work of Martinez-Mateu et al. (2018) evaluated how electrode-endocardium distance, far-field sources, and inter-electrode distance affect the accuracy of localizing rotors by using a virtual basket catheter. In their simulation study, basket catheter-based phase mapping (PM) successfully detected rotors even when the basket was not in full contact with the endocardial wall, but many phantom rotors were also detected. Despite the different types of catheter analyzed in Martinez-Mateu et al. (2018) their overall message was in accordance with our observations: (1) the basket catheter resulted positioned

preferably closer than 0.5 cm to the atrial tissue; (2) increasing the electrode density improved rotor detection if the basket was not located close to the rotor area and (3) accuracy varied depending on the position of the basket inside the atrial cavity and the number of electrodes. In our study, in any simulated condition, we did not detect any phantom rotor, a fact which may be due to the interpolated basket phase maps used in Martinez-Mateu et al. (2018). Indeed, the lack of false detections in our study could be due to the optimization of the LAT detection step compared to the standard PM which relies only on the Hilbert transform. Similarly, the very recently proposed Direct Graph Mapping (DGM) (Van Nieuwenhuyse et al., 2021) technique outperformed the PM approach, by being able to exclude some of the false rotors detected in Martinez-Mateu et al. (2018).

In Martinez-Mateu et al. (2019), the same group assessed the effects of the multi-electrode systems on the accuracy of rotors’ localization by varying the multi-electrode array configuration. They analyzed rotors’ detection by varying electrode-to-tissue distance (d) from 0.9 to 19.8 mm and inter-electrode distance (IED) from 0.9 to 18 mm. Results showed that increasing the distance from the wall decreased the sensitivity. Surprisingly, the effects of increasing IED on the phase maps seem to be stronger when the electrode is closer to the tissue. Simulation results suggest that an inter-electrode distance of less than 9 mm was a sufficient spatial resolution to detect rotors with relatively high sensitivity, for low distances to the tissue (d = 0.9 mm). However, when the spatial resolution of the electrodes was poor, rotors’ detection improved by increasing the electrode-to-tissue distance. This result was in discordance with our discoveries that increasing the inter-electrode distance (from 3 to 6 mm) causes the incapability of detecting rotors at a closer distance (2 mm) from the wall. Indeed, they tested a larger inter-electrode distance (up to 18 mm), compared with us, and it can be observed that for IED > 9 mm the sensitivity was very low, proving that the detection was almost lost.

A few recent studies (Ganesan et al., 2020; Ravikumar et al., 2021) aimed at validating new algorithms for rotor detection, and simulated EGM acquisition using different mapping catheters.

Ganesan et al. (2020) have described a mapping algorithm that delineated the area of meandering repeating-pattern AF sources suggesting an optimal position for the subsequent catheter location. This new algorithm was validated using EGMs acquired with two catheters (Lasso and spiral catheters) at different electrode-wall distances and simulating non-optimal contact conditions by making electrograms randomly isoelectric. They found a high sensibility of their results to the presence of poor catheter contact: as the percentage of the missing electrodes increased (already at 5%), the algorithm success gradually decreased for both Lasso and spiral configuration. The latter one was more robust toward missing electrodes. Due to the different types of catheters employed in Ganesan et al. (2020), a direct comparison with our results is difficult. However, notably, both HDGrid and PentaRay catheters we simulated were more robust, being able to detect the AF driver with 50% of non-contact electrodes and with a much smaller estimation error compared to the resolution of the method proposed in Ganesan et al. (2020) for defining a successful detection.

Lately, Ravikumar et al. (2021) tested the performance of several novel techniques developed to identify rotor pivot points in the case of multielectrode multispline (total of 160 signals) and grid catheter-based (total of 96 signals) sequential mapping for stationary rotors. Results in these two realistic clinical scenarios confirmed the capability of identifying the core of the stationary rotor with high sensitivities. Unfortunately, no simulation to test the effect of the catheter-LA wall distance was performed.

Our study represents a systematic analysis based on computer simulations of the most frequently used mapping catheters in clinical practice to evaluate the influence of inter-electrode spacing, catheter coverage, and endocardium-catheter distance for rotor detection. Even if some of our results confirm what was already partially reported in other studies applying different catheters, this is the first attempt to make a detailed and comprehensive evaluation of the most used mapping catheter design and acquisition condition which may affect a correct rotor detection. The computational environment we developed provides motivation for future clinical validation.

## Study Limitations

The presented tool shows some limitations.

The first limitation is relies on the simplification of the atrial tissue to a plane, neglecting the anatomical complexity, heterogeneous fiber orientation, and tissue anisotropy. A preliminary test on a real anatomical atrial shape (see Supplementary Figure 2), acquired during ablation in a patient, showed that the rotor detection was successful also in this realistic setup. Moreover, AF mechanisms can lead to spatially heterogeneous alterations of conductive and ionic properties, but we had to focus on a simple set of homogeneous electrophysiology and remodeling data. In addition, clinical EGM signals are contaminated by far-field effects from the ventricles and noise which reduce the quality in comparison with our simulated EGMs. So, since the real EGMs during AF are more complex and with a higher number of artifacts, those simulated here may have reduced the reliability of the computed phase maps. In addition, in our approach white Gaussian noise was used as a standard approach to mimic broadband measuring noise. As far as we know, the value of such noise in real EGMs is not known. In order to test the impact of our choice we tested several levels of SNR (from 50 to 20 dB) (see Supplementary Figure 2 and Supplementary Table 1) with the catheter located at 2 mm from the LA wall, a condition in which the detection of the rotor was critical with a 50 dB SNR. Results showed the algorithm fails rotor detection when SNR decreases to 20. It is worth noting that our main conclusion was how catheter geometry and specific parameters affect its capability to detect rotors: given a certain, quite “typical,” activation pattern we have quantified the impact of changing catheter position and parameters, therefore focusing on “differential” effects, which are very likely conserved even in our simplified model.

Regarding cardiac tissue simulation, the value of the diffusion coefficient was based on previous studies (Bai et al., 2019); nevertheless, this choice resulted in a lower CV than realistic CVs in human atria in AF (e.g., 53 ± 12 cm/s; Konings et al., 1994). Other parameters such as spatial resolution may minimally affect CV (Wilhelms et al., 2013). Therefore, additional tests with an increased value of the diffusion coefficient and spatial resolution should be performed to simulate more realistic conduction velocities, whilst increasing spiral wave propagation smoothness, but we do not expect these improvements in the tissue simulation to affect the findings of our study.

We presented simulations for a single scenario of a rotor area without additional wave breaks; this could limit our ability to extrapolate quantitatively the results to more complex fibrillatory wave propagation scenarios. While a comprehensive analysis of all the propagation patterns that can be generated by the different mechanisms initiating and sustaining AF goes beyond the scope of the present work, in order to enforce the applicability of our approach we simulated the mapping of a target (breakthrough) pattern (see Supplementary Video 4) with both 4 × 4 HDGrid and PentaRay 4-4-4. In that case, our tool, which was specifically designed to detect phase singularities, in fact did not detect any rotor.

The reliability of the proposed approach cannot be easily assessed without a comparison with either experimental data or a more sophisticated computational model. Unfortunately, real-life data cannot be used for the evaluation of the proposed tool without the actual location of AF drivers being known. Remarkably, a stringent validation would require very complex surgical or ex-vivo experiments with simultaneous high density (e.g., optical) mapping and catheter acquisitions. This kind of setup would be feasible only epicardially, and would be definitely beyond the scope of the present study. A first attempt to simulate 3D complex geometries in which the concavity of the LA surface may not allow a perfect contact between the mapping catheter and the LA endocardial wall was tested by considering detached electrodes. These preliminary tests should be considered as a step forward toward the simulation of a more complex 3D geometry.

## Conclusion

The computational framework we developed was based on realistic catheter shapes designed with parameter configurations that resemble clinical settings. It allowed the simulation of both commercial high-resolution catheters and catheters in which parameters such as the number of electrodes, inter-electrodes distance, and endocardium-catheter distance were changed. Results showed that it is well suited to investigate how mapping catheter geometry and location affect AF driver detection, therefore making it a reliable tool to design and test new mapping catheters. In addition, it provided visual feedback about the spatial density map of the rotor tip trajectory, a proposed marker of the presence of an AF source.

## Data Availability Statement

The data and the software are available upon request to the authors. The inquiries can be directed to the corresponding author.

## Author Contributions

CB: simulations, analysis of the results, manuscript drafting, and figure preparation. CF: software development, manuscript drafting, and video preparation. CT and PS: data acquisition and revision of the manuscript. SS and CC: conception of the study, software development, analysis of the results, and revision of the manuscript. All authors contributed to the article and approved the submitted version.

## Conflict of Interest

The authors declare that the research was conducted in the absence of any commercial or financial relationships that could be construed as a potential conflict of interest.

## Publisher’s Note

All claims expressed in this article are solely those of the authors and do not necessarily represent those of their affiliated organizations, or those of the publisher, the editors and the reviewers. Any product that may be evaluated in this article, or claim that may be made by its manufacturer, is not guaranteed or endorsed by the publisher.
