# Supplementary material for: Computational Analysis of Mapping Catheter Geometry and Contact Quality Effects on Rotor Detection in Atrial Fibrillation
Source: Front Physiol. 2021 Dec 9;12:732161. doi: 10.3389/fphys.2021.732161 (PMC8696082; doi:10.3389/fphys.2021.732161)
Supplement: Supplementary file 1 [file Data_Sheet_1.PDF]

Supplementary Material

1 Supplementary Figures

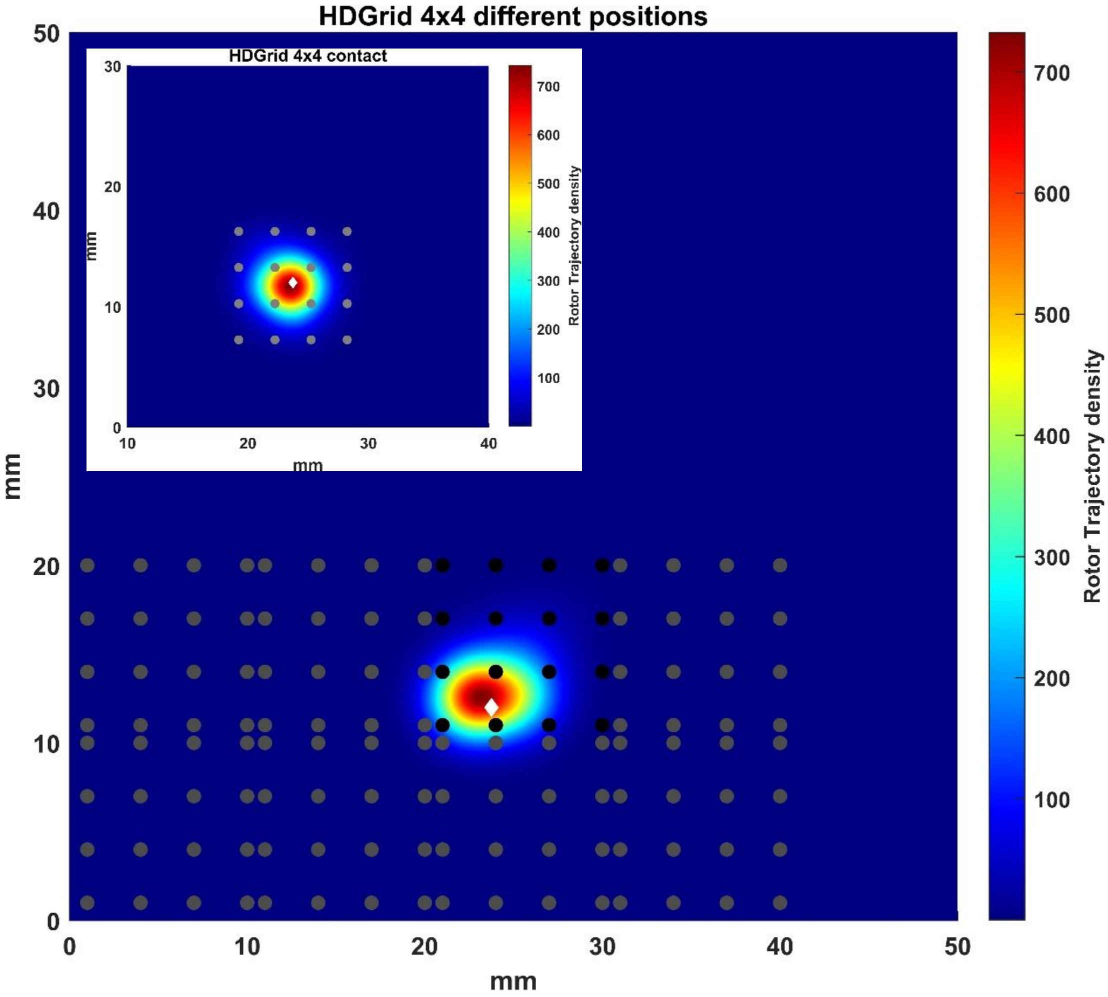

Supplementary Figure S1: Density maps of rotor tip trajectory reconstructed by moving the 4x4 HD Grid catheter with an inter-electrode distance of 3mm in 8 different positions. The black catheter position is the one that detected the rotor. The inset shows the same result as Figure 3, panel B.

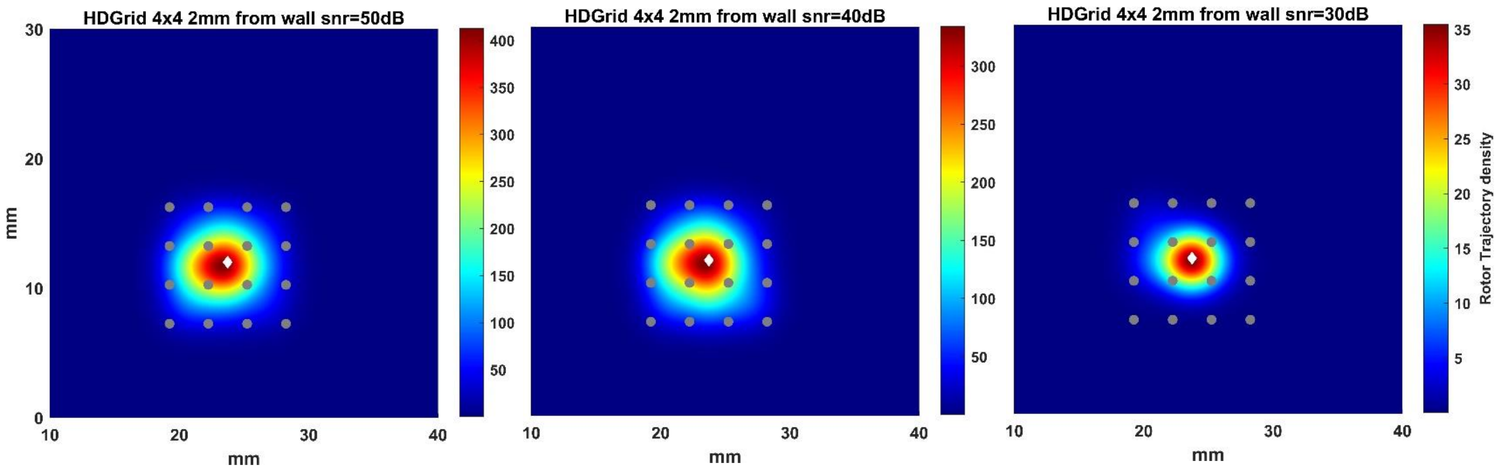

Figure S2: Density maps of rotor tip trajectory reconstructed with an inter-electrode distance of 3mm, with a 4x4 HD Grid catheter at 2mm distance from the atrial wall with different noise levels (A-50dB, B-40dB, C-30dB). The patch has been zoomed (x-axis between 10 and 40mm, y-axis between 0 and 30mm). Colour encodes the trajectory density value. The white diamond marker represents the rotor tip position of the ground truth.

Table S1. Simulation parameters and resulting indexes derived from the phase singularity map for HDGrid 4x4 at 2mm wall distance with different noise levels (50-40-30dB, and 20dB for which the rotor was not found).

	**Inter-electrode distance (mm)**	**Noise level (dB)**	**Rotor duration (s)**	**Density Peak**	**Standard Deviation (mm)**	**Peak to peak distance between ground truth and estimated rotor (mm)**
Ground Truth			7.75	544	8.23	
HD Grid 4x4	3	50	6.7	413	2.54	0.25
		40	5.8	334	2.7	0
		30	0.33	35	1.5	0.25
		20	-	-	-	-
